# Differential Activity of Antioxidants in Testicular Tissues Following Administration of *Chlorophytum borivilianum* in Gamma-Irradiated Swiss Albino Mice

**DOI:** 10.3389/fphar.2021.774444

**Published:** 2022-01-17

**Authors:** Ruchi Vyas, Kavindra Kumar Kesari, Petr Slama, Shubhadeep Roychoudhury, Rashmi Sisodia

**Affiliations:** ^1^ Department of Zoology, S.S Jain Subodh PG College, Jaipur, India; ^2^ Department of Zoology, University of Rajasthan, Jaipur, India; ^3^ Department of Applied Physics, School of Science, Aalto University, Espoo, Finland; ^4^ Department of Animal Morphology, Physiology and Genetics, Faculty of AgriSciences, Mendel University in Brno, Brno, Czech Republic; ^5^ Department of Life Science and Bioinformatics, Assam University, Silchar, India

**Keywords:** irradiation, oxidative stress, *C. borivilianum*, glutathione, glutathione-s-transferase, catalase, testicular tissue

## Abstract

**Background:** Oxidative stress induced by radiation causes variable expression of antioxidant enzymes in a tissue-specific manner. Testicular tissues carry out the complex process of spermatogenesis, and studies indicate that testicular damages due to irradiation require long-term recovery before complete resumption. Ionizing radiation also causes oxidative stress in tissues, leading to testicular damage. Aims and Objectives: This study measured differential expression of antioxidant enzymes following administration of *C. borivilianum* root extract (CRB) in response to irradiation-induced oxidative stress. The activity of various important endogenous enzymatic defense systems was evaluated and correlated for strength of association.

**Materials and method:** Two forms of *C. borivilianum* (CB) extracts [CB alone and CB-silver nanoparticles (AgNPs)] were administered at a dose of 50 mg/kg body weight to Swiss albino male mice for 7 consecutive days. After that, they were irradiated with 6 Gy irradiation and further used to study various parameters of antioxidant enzymes.

**Results:** Results indicate a significant increase in the level of glutathione (GSH) and the activity of GSH-related antioxidant enzymes in irradiated mice treated with CRE and CRE-AgNPs (silver nanoparticles biosynthesized using *C. borivilianum* root extract) in comparison to non-pretreated ones (groups I and II). Reciprocal elevation was observed in related enzymes, that is, glutathione S-transferase activity (GST), glutathione reductase (GR), and glutathione peroxidase activity (GPx). Elevation in the activity of catalase (CAT) and superoxide dismutase (SOD) was also evident in both the irradiated groups pretreated with CRE-AgNPs. However, expression of CAT in the CRE-treated irradiated group was similar to that of the non-treated irradiated group. Higher association among CAT-SOD, CAT-GPx, and GR-GST was observed.

**Conclusion:** Overall, it was observed that testicular cells post-irradiation in all groups go through intense oxidative stress; however, groups pretreated with CRE or CRE-AgNPs indicated better toleration and resumption of antioxidant capacity. CRE or CRE-AgNPs pretreated non-irradiated groups mostly remained within the control range indicating stimulated expression of antioxidants.

## 1 Introduction

Ionizing radiation induces oxidative stress, and, therefore, can cause severe damage to tissues and genetic materials. Energy formed due to ionizing radiation can make harmful changes in the DNA, and if not repaired, it may result in cell death or mutation. Ionizing radiation is found everywhere nowadays as it is highly beneficial in ensuring hygiene, treatment of cancer, smoke detectors, *etc*. At low doses, radiation exhibits some beneficial effects in reducing oncogene-induced carcinogenesis (Kim et al., 2015). Most apparent risk of ionizing radiation exposure is through diagnostic medical examinations. There are apprehensions that ionizing radiation in diagnostic imaging is an initiator of carcinogenesis (CTRDMDP, 2011). A study by Mettler et al. (2000) assumed that the theoretical risk of fatal cancer from the first CT scan for a dose of 10 mSv is estimated to be nearly 1 in 2000. In another way, CT examination is ∼100 folds higher than X-ray examination; thus, risk of cancer from CT scan is higher than that from X-ray diagnosis.

Increase of endogenous reactive oxygen species (ROS) is one of the important factors that damage the body. Although ROS benefit physiological processes, such as removal of infectious pathogens, healing, and repair of tissues (Tovar-y-Romo et al., 2016; Cavaillon, 2018), nevertheless, excessive levels can damage tissues (Yu et al., 2015; Scialo et al., 2017). When exposed to ionizing radiation for treatment, it does not only direct toward tumor cells but also affects the surrounding microenvironment (Haimovitz-Friedman et al., 2020). Indirect unspecified effects of ionizing radiation may result in inflammatory responses (Fuks et al., 1995; Paris et al., 2001; Mizrachi et al., 2016). Moeller et al. (2004) indicated that ROS burst following radiation generate waves of hypoxia/re-oxygenation.

Plants have a wide variety of bioactive compounds that adapt to the constantly evolving environment. A previous study provides strong evidence that some antioxidants in plants can efficiently scavenge oxidative radicals and, thus, can protect from various diseases (Hassan et al., 2017). A recent review on the role of plants in radioprotection indicated that crude or fractionated extracts, phytocompounds, and polysaccharides can provide protection from ionizing radiation (Dowlath et al., 2021). This study aimed to evaluate the radioprotective effect of *Chlorophytum borivilianum*.

*C. borivilianum* Santapau and Fernandes (family: Asparagaceae; sub family: Anthericoideae) (Santapau and Fernandes, 1955; Govaerts, 1999) is known for its medicinal property against various conditions such as inflammation or antimicrobial/antifungal (Parveen et al., 2020), infertility, stress (Devi et al., 2021), and cirrhosis (Singh et al., 2011; Tripathi et al., 2019; Kaur and kaur, 2020). Recently, it has gained a well-established national and international market for being the herbal alternative of “Viagra” without any side effects. Various studies showed that aqueous extract of dried roots of *C. borivilianum* enhances the sexual arousal, vigor, and libido in Wistar rats along with a significant increase in sperm count (Kenjele, et al., 2008). Surprisingly, although *C. borivilianum* has been used for ages by the herbal medical practitioners, the amount of research conducted to understand its effects remains considerably low.

*C. borivilianum* has been used for over 4,000 years, according to the Hindu epic Srimad Bhagawat. It belongs to the “Vajikarana Rasayana” group of Ayurvedic plants, which are utilized for rejuvenation (Miraj et al., 2020; Verma et al., 2020) and revitalization characteristics in order to improve sexual dynamics (Puri, 2003) and alleviate sexual dysfunction. This is also the basis for Kamasutra’s medical recommendations (Hooper, 2002). An NCBI search result for the name “*Chlorophytum borivilianum*” found only 200 results. Among those, most of the studies were only of botanical importance (data retrieved on 08–11–2020). Moreover, the Botanical Survey of India has identified it as an endangered species in their “Red Data Book of Indian Plants.” It is predicted that the Indian forests may lose this valuable plant if conservation measures are not adopted (Tripathi et al., 2012). Thus, due to high and diversified commercial importance, it has been instigated to reserve in the list of “threatened plants category.” Interestingly, biosynthesized nanoparticles (CRE-AgNPs) not only decrease the amount of dose required for the treatment but can also help to reduce the day-to-day growing demand of it.

Therefore, considering the importance of this plant and owing to environmental radiation and other lifestyle disorders, natural radioprotectors are the need of the hour. In this study, benefits of natural radioprotectors and nanoparticles are combined, thus amplifying its positive effects. Furthermore, this study examines alterations, if any, in the levels of antioxidative enzymes in testicular tissues of mice following spells of gamma radiation. Results of these were compared with the level of antioxidant enzymes in mice following administration of crude *C. borivilianum* root extract (CRE) and the same extract tagged with silver nanoparticles (CRE-AgNP) against radiation to examine potential amelioration. Moreover, to date, there is no report on the biosynthesis of AgNPs using the *C. borivilianum* root extract and their use as an antioxidant.

## 2 Materials and Methods

### 2.1 Preparation of *C. borivilianum* Root Extract

*C. borivilianum* root extract (CRE) was obtained commercially in this study. However, it was identified (RUBL No. 19902) from the Department of Botany, the University of Rajasthan, Jaipur, India, and also recognized as a plant of edible characteristics (Sharma and Kumar 2012). In this study, a concentration of 1–5% was prepared by mixing CRE into 250 ml of deionized water. The extract was then filtered twice by using the Whatman No. 1 filter paper to obtain a clear solution. This clear solution was then refrigerated at 4°C in a 250-ml Erlenmeyer flask until further use. The complete process of preparation of the extract was maintained under sterile conditions.

### 2.2 Synthesis of Silver Nanoparticle Tagged CRE

5 ml CRE was mixed with 95 ml of AgNO_3_^(−)^ for synthesis of tagged molecules. Confirmation of formation of CRE-AgNPs was ensured by the development of reddish brown color (α-β) (Christensen et al., 2011). Morphological and structural attributes were identified and recorded previously by transmission electron microscopy (TEM) (Singh et al., 2018).

### 2.3 Test Animals

Swiss albino mice (*Mus musculus*) (6–8 weeks), weighing between 20 and 30 g, were used for the present experiment. These random-bred male mice were maintained under continuous observation of the departmental facility. Animals were maintained at 24 ± 3°C with 12-h light and 12-h dark periods. Commercially obtained pellet diet (Ashirwad Pvt. Ltd., India) was provided to the experimental animals. Drinking water was provided *ad libitum*. The ethical committee of the Department of Zoology, Centre for Advanced Studies (CAS), the University of Rajasthan, Jaipur, India, has approved the experimental protocol (IAEC Approval No. 1678/90/re/S/12/CPCSEA dated June 16, 2017). All the experiments were performed according to ethical guidelines.

### 2.4 Experimental Design

Based on our previous study, an optimal dose of CRE and CRE-AgNPs was decided (Singh et al., 2018), and the characterization and optimization of CRE-AgNPs were also performed (Singh et al., 2018). Alterations in the activity of antioxidant enzymes were observed in testicular tissues following gamma radiation. Comparative differentiation was made between antioxidant activities in animals based on pretreatment with CRE, with CRE-AgNPs, and with respective controls.

Pretreatment with CRE and CRE-AgNPs was continued for 7 days, following which mice were irradiated with 6 Gy gamma radiation. Briefly, the Cobalt Teletherapy Unit (ACT-C9) (Bhabhatron-II TAW telecobalt machine) at the Cancer Treatment Center, Radiotherapy Department, SMS Medical College and Hospital, Jaipur, India, was used for irradiation. Unanesthetized animals were restrained in a well-ventilated perspex box, and the whole body was exposed to gamma radiation. Dosimetry was then calculated as 1.07 Gy/min from the source to surface distance of 80 cm.

**FIGURE 1:**
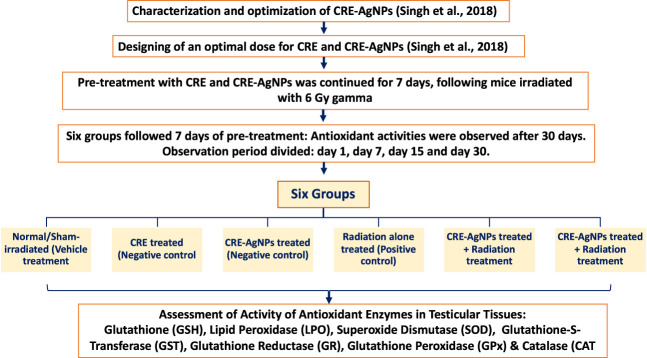
Illustration of experimental design and groups along with measurement parameters.

Six groups were formed constituting the sham control, negative control, positive control, and test groups. Following 7 days of pretreatment, antioxidant activities were observed for 30 days. The period of observation was further divided into four parts, that is, day 1, day 7, day 15, and day 30. On each scheduled observational day, 5 animals from each group were sacrificed by cervical dislocation. Experimental groups consisted of 20 animals each, and the overall design of the study is presented in Figure 1.

Group 1: Normal/Sham-irradiated (vehicle treatment): This group was given double distilled water (DDW) through oral gavages once in a day for 7 consecutive days (dose equivalent to CRE).

Group 2: CRE treated (negative control): This group was treated with 50 mg/kg body weight of CRE dissolved in distilled water through oral gavages for 7 consecutive days once daily.

Group 3: CRE-AgNPs treated (negative control): This group was treated with 50 mg/kg body weight of CRE-AgNPs dissolved in distilled water for 7 consecutive days once daily.

Group 4: Radiation-alone treated (positive control): This group was given double distilled water (DDW) through oral gavages once in a day for 7 consecutive days. On the 7^th^ day, mice were irradiated with 6 Gy gamma radiation.

Group 5: CRE treated + radiation treatment: This group was treated with 50 mg/kg body weight of CRE dissolved in distilled water through oral gavages for 7 consecutive days once daily. On the 7^th^ day, mice were irradiated with gamma radiation (6 Gy).

Group 6: CRE-AgNP treated + radiation treatment: This group was treated with 50 mg/kg body weight of CRE-AgNPs dissolved in distilled water through oral gavages for 7 consecutive days once daily. On the 7^th^ day, mice were irradiated with 6 Gy gamma radiation.

### 2.5 Assessment of Activity of Antioxidant Enzymes in Testicular Tissues

The tissues were thawed and homogenized in 10% w/v ice-cold 0.05 m potassium phosphate buffer (pH 7.4). In total, 0.2 ml of the homogenate was used for TBARS estimation, and 1.0 ml of the homogenate was mixed with 10% trichloroacetic acid 175 (TCA) and centrifuged for tissue GSH estimation. The remaining homogenate was centrifuged at 40,000 × *g* for 60 min, and the supernatant was used for the estimation of superoxide dismutase (SOD) and catalase (CAT). Protein concentration was estimated according to Bradford (1976).

#### 2.5.1 Glutathione

Reduced glutathione was measured fluorometrically using the method of Hissin and Hilf (1976. Then a 250-mg testicular cell pellet was suspended in a medium of 25% metaphosphoric acid and potassium phosphate buffer (pH 8.0), and sonicated for 10 min. Then it was centrifuged at 30,000 × g for 30 min. GSH assay fluorescence was determined with a fluorescence spectrophotometer at 420 nm (excitation at 350 nm) after incubating 0.2 ml of supernatant with 1.7 ml of potassium phosphate-EDTA buffer (pH 8.0) and 0.1 ml of fluorescence reagent *o*-phthaldialdehyde (1 mg/ml) for 15 min. The result of assays was calculated against a standard calibration curve for GSH.

#### 2.5.2 Lipid Peroxidase

Lipid peroxidation (LPO) levels were measured by the thiobarbituric acid (TBA) reaction, following the method of Ohkawa et al. (1979). Tissue supernatants (50 μL) were added to test tubes containing 2 μL of butylated hydroxytoluene (BHT) in methanol. Next, 50 μL of an acid reagent (1 M phosphoric acid) was added, and finally, 50 μL of the TBA solution was added. The tubes were mixed vigorously and incubated for 60 min at 60°C. The mixture was centrifuged at 10,000 × *g* for 3 min. The supernatant was put into wells on a microplate in aliquots of 75 μL, and its absorbance was measured with a BIO RAD Benchmark Plus plate reader at 532 nm. TBARS levels were expressed as nmol/mg tissue.

#### 2.5.3 Superoxide Dismutase

The level of SOD was estimated (Marklund and Marklund, 1974). A weight of 2.85 g of Tris and 1.11 g of EDTA-Na2 were dissolved in 1 L of distilled water. A weight of 0.252 g of pyrogallol was dissolved in a solution of 0.6 ml of concentrated hydrochloric acid diluted in 1 L of distilled water. Spectrophotometer was adjusted to read zero using the Tris-EDTA buffer. Control and sample test tubes were prepared and then pipetted into test tubes. Absorption was read at the wavelength of 420 nm against the Tris-EDTA buffer at zero time and after 1 min of the addition of pyrogallol. One unit of SOD activity is defined as the amount of enzyme required to cause 50% inhibition of pyrogallol autoxidation.

#### 2.5.4 Glutathione-S-Transferase

The GST activity was measured by the method as described by Habig et al. (1974). The reaction mixture containing 1 ml of buffer, 0.1 ml of 1-chloro-2, 4-dinitrobenzene (CDNB), 0.1 ml of homogenate, and 1.7 ml of distilled water was incubated at 37°C for 5 min. The reaction was then started by the addition of 1 ml of glutathione. The increase in absorbance was followed for 3 min at 340 nm. The reaction mixture without the enzyme was used as the blank.

#### 2.5.5 Glutathione Reductase

Glutathione reductase was assayed according to the method of Beutler and West (1984). Glutathione reductase catalyzes the reduction of oxidized glutathione (GSSG) by NADPH or NADPH to reduced glutathione. The activity of the enzyme was measured at 340 nm following the oxidation of NADPH. GR is a flavin enzyme, and it has been found that it was not fully activated by FAD in normal samples. Complete activation of apoenzyme requires the pre-incubation of the enzyme with FAD. This is done prior to the addition of GSSG or NADPH to the reaction system.

#### 2.5.6 Glutathione Peroxidase

GPx activity was determined according to the method given by Wood (1970). It was assayed using a 1-ml cuvette containing 700 μL of phosphate buffered solution (75 mm; pH 7.0) to which the following solutions were added: 25 μL of glutathione reductase solution (100 μ/mL), 25 μL of sodium azide (0.12 m), 50 μL Na_2_ EDTA (0.15 mm), 50 μL of β NADPH (3 mm), and 50 μL aliquots of supernatant obtained after centrifugation at 14,000 × g for 25 min. The above reaction mixture was equilibrated at 25°C after mixing thoroughly, and the reaction was initiated by the addition of H_2_O_2_ (7.5 mm). The decrease in absorbance was read at 340 nm at 1 min interval for 5 min for the conversion of NADPH to NADP. The enzyme activity was expressed as nmol NADPH oxidized/min/mg protein using 6.22 mm^−1^ cm^−1^ as the molar extinction coefficient.

#### 2.5.7 Catalase

The catalase activity was determined according to the U.V. assay method of Aebi (1974). In this method, 0.1 ml of hemolysate was added to 5 ml of water, mixed, and allowed to stand for 5 min. In a duplicate, 1 ml of phosphate buffer was added to 2 ml of hemolyte, 1 ml of hydrogen peroxide (30 mm) solution was added after the spectrophotometry, and the absorbance was monitored at 750 nm for 3 min.

### 2.6 Statistical Analysis

Numeric values obtained from parametric evaluation for each group were expressed as mean ± SE. One-way ANOVA along with Tukey’s multiple comparison test was carried out to evaluate variance (MINITAB, Pennsylvania, United States). *p* < 0.05 was considered significant variance. Surface plots were used to represent high and low peaks in activities of antioxidant enzymes. Comparative differences in activities of antioxidant enzymes were measured among day 1, day 7, day 15, and day 30 by stock plots (EXCEL, Microsoft, United States). Pearson’s correlation was applied to identify strength of association between variables of individual antioxidant activity against irradiation-induced oxidative stress.

## 3 Results

Antioxidant enzyme responses were separated into two parts for better evaluation of oxidative stress, that is, groups 1–3 (non-irradiated) and groups 4–6 (irradiated). Due to extremely significant alterations in antioxidant activities following irradiation, the level of significance in partitioned groups was measured against group 1 and group 4 for non-irradiated groups and irradiated groups, respectively. In most cases, CRE- and CRE-AgNP–treated irradiated groups remained significantly below the control range (group 1) (*p* < 0.05), and therefore, activities of those antioxidants which resumed back to the control range are mentioned in the text.

### 3.1 Glutathione Response

**FIGURE 2:**
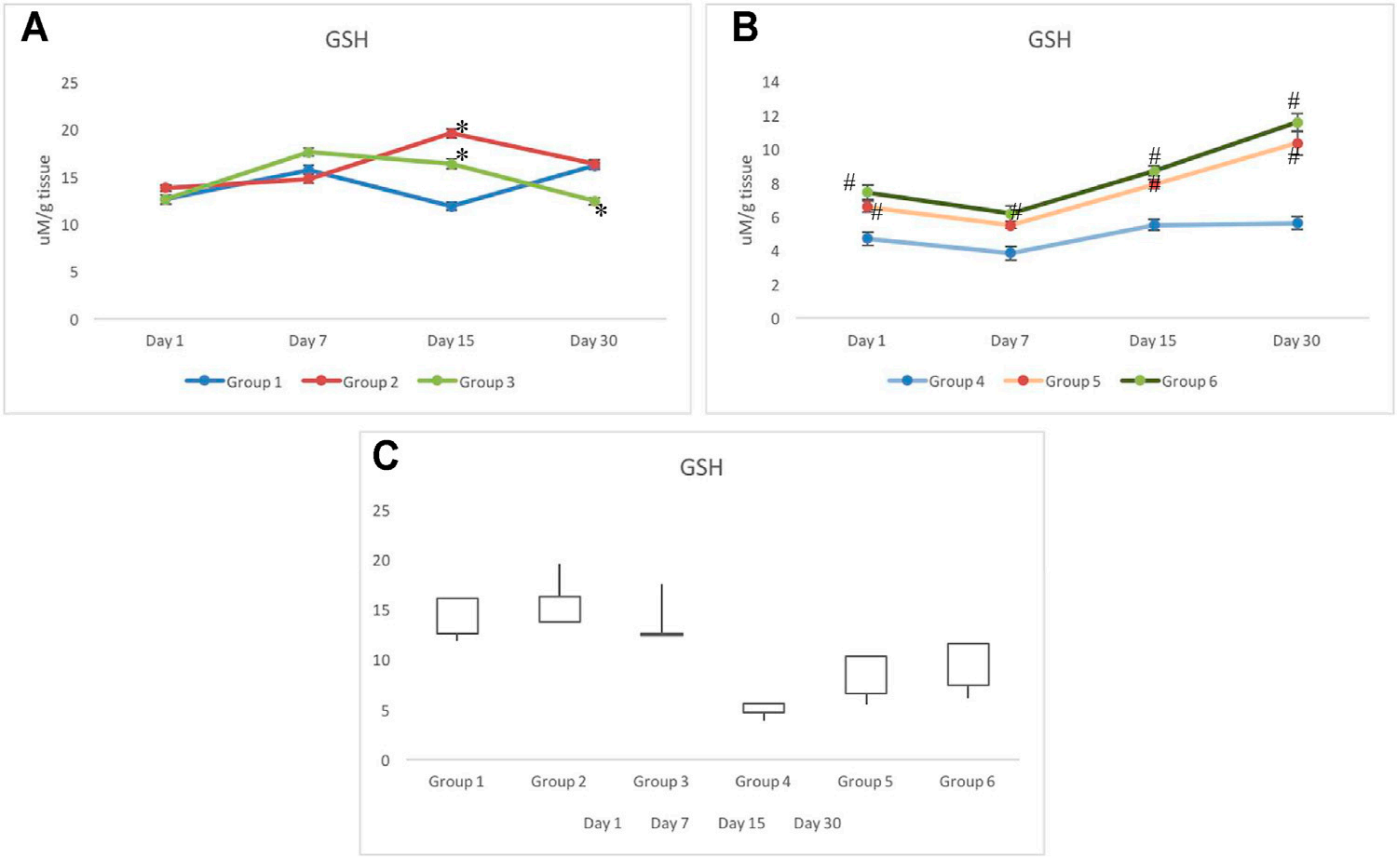
Response of reduced glutathione in non-irradiated **(A)** and irradiated **(B)** groups pretreated with CRE/CRE-AgNPs/sham. Gain/loss in the activity of glutathione has been noted and presented through the box plot **(C)**. * *p* < 0.05 significance level was measured against group 1. ^**#**^ *p* < 0.05 significance level was measured against group 4.

The activity of GSH reflected no alteration on day 1 in groups 1–3. However, as time progressed to day 7, a slightly higher level of GSH was observed in group 3 (17.57±0.46 μM/g tissue) than in group 1 (15.69±0.47 μM/g tissue) and group 2 (14.74±0.46 μM/g tissue). On day 15, deviation in the level of GSH was clearer, as group 2 indicated the highest level of GSH, and was recorded as 19.54±0.44 μM/g tissue. However, the GSH level in group 3 was recorded as 16.33±0.50 μM/g tissue against 11.84±0.43 μM/g tissue of group 1. On day 30, elevation in the level of GSH was observed in group 1 which was recorded as 16.13±0.39 μM/g tissue and was similar to that of group 2 (16.31±0.44 μM/g tissue). However, the level of GSH in group 3 declined further to 12.41±0.39 μM/g tissue (Figure 2A).

In groups 4–6, radiation indicated substantial impact on the level of GSH on day 1 and day 7. Similar patterns were observed in all three groups exposed to radiation (Figure 2B). However, the level of GSH depletion in groups 5–6 was at least 30–40% lower than that in group 6 on day 1. Increase in the level of GSH was observed in both groups administered with CRE and CRE-AgNPs, but the rate of increase was measured to be higher in group 6 than that in group 5 (Figure 2B). However, despite the increase in GSH levels in group 5 and group 6, on day 30, the level of GSH remained significantly lower than that of the control group (Figure 2C).

### 3.2 Lipid Peroxide Response

**FIGURE 3:**
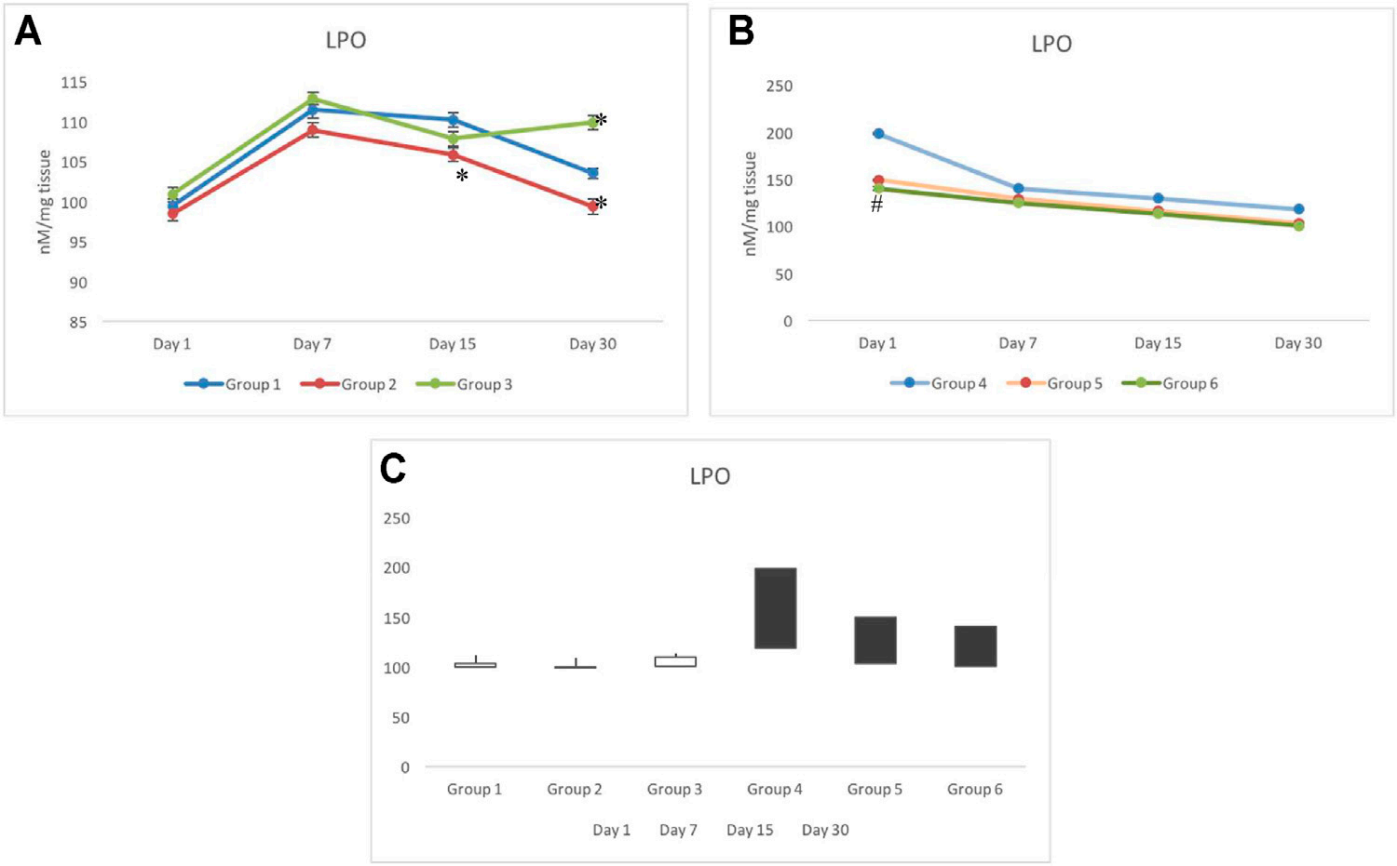
Variation in lipid peroxidation (LPO) in non-exposed **(A)** and exposed groups **(B)**. The stock plot indicates highest and lowest peroxidation levels from day 1 to day 30 **(C)**. The white box indicates the gain in peroxidation, whereas the black box indicates the loss in peroxidation. *p < 0.05 significance level was measured against group 1. ^**#**^ *p* < 0.05 significance level was measured against group 4.

Following withdrawal from the treatment of CRE (group 2) and CRE-AgNPs (group 3), on day 1, all non-exposed groups responded similar to that of the control group (group 1). However, following 7 days of withdrawal, a slight deviation between the groups was observed where group 1 to group 3 remained close (111.42±0.99 nM/mg tissue and 112.87±0.83 nM/mg tissue, respectively), and significant decline in the level of LPO was observed in group 2 (108.9±0.89 nM/mg tissue). On day 15, group 3 also depleted further to get close to group 2 and was recorded as 107.85±0.89 nM/mg tissue. On day 30 of withdrawal from treatment, a clear deviation was noted in groups 2 to 3 with respect to the control. A slight elevation in the level of LPO was recorded in group 3 with respect to the control, whereas, a gradual decline in the level of LPO was noted in groups 1 to 2 (Figure 3A).

Unlike in non-exposed groups 1 to 3, deviation in the level of LPO was only observed on day 1 in radiation exposed groups (groups 4–6) where group 4 was noted with the highest level of LPO (198.31±1.15 nM/mg tissue), groups 5 and 6 remained close to each other (149.23±0.68 nM/mg tissue and 140.17±1.8 nM/mg tissue, respectively) and significantly lower than group 4. On days 7–30, a steep decline in groups 4 to 6 was observed (Figure 3B). During 7, 15, and 30 days of investigation, a gradual decline in the levels of LPO was observed in groups 5 and 6 with respect to group 4. The level of LPO in groups 5 and 6 completely resumed back to the control (group 1) range, whereas, despite a significant decline in group 4 on day 30, it remained significantly higher than the control group (group 1). The stock plot revealed that despite the highest level of LPO on day 30, group 4 indicated maximum depletion in the level of LPO from days 1–30, followed by group 5 and group 6 (Figure 3C).

### 3.3 Superoxide Dismutase Response

**FIGURE 4:**
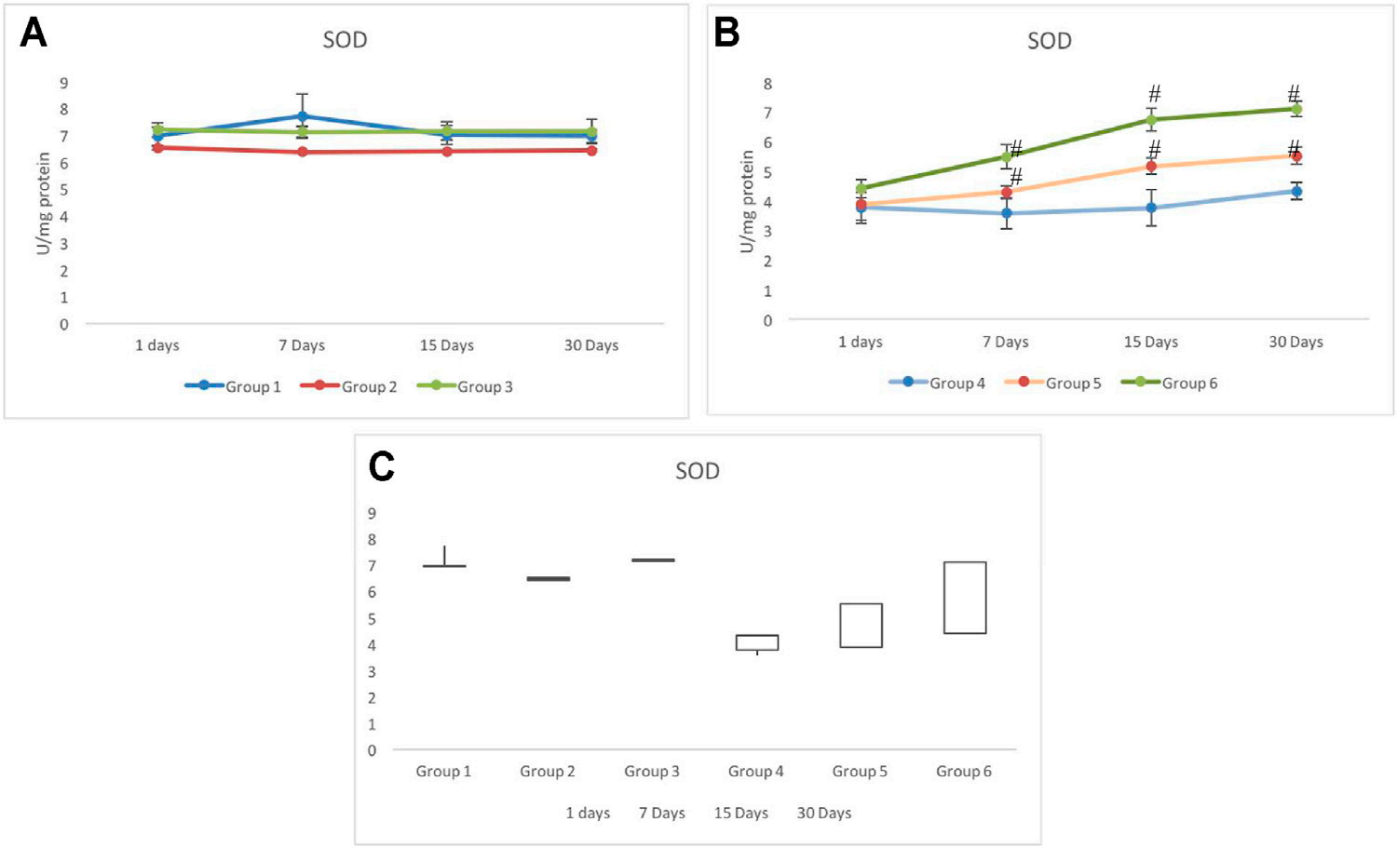
Alteration in the activity of superoxide dismutase (SOD) in CRE/CRE-AgNP pretreated animals. **(A)** Non-irradiated groups. **(B)** Irradiated groups. **(C)** Gain/loss in the activity of superoxide dismutase. *p < 0.05 significance level was measured against group 1. ^**#**^ *p* < 0.05 significance level was measured against group 4.

Non-exposed groups 1 to 3 showed relatively similar response for the activity of SOD in testicular cells. However, a slightly lower but stable activity was observed in group 2 with respect to group 1 (Figure 4A).

Main changes in the activity of SOD were observed in the irradiated groups. Although, on day 1, similar activities were recorded in all the three irradiated groups (4–6), by day 7, a clear deviation was recorded in all three groups. The lowest SOD activity was observed in group 4 (3.58±0.52 U/mg protein), whereas the maximum activity was observed in group 6 (5.49±0.41 U/mg protein) on day 7. All the three exposed groups indicated a gradual increase in the activity of SOD from day 7 to day 30. Group 5 indicated a significantly higher activity than group 4. Group 6 maintained a maximum activity from day 7 to day 30. However, the SOD activity on day 30 was still lower than that of the control (group 1) (Figure 4B). The stock plot indicated a maximum gain in the SOD activity in group 6 followed by group 5 and group 4 (Figure 4C).

### 3.4 Glutathione-S-Transferase Response

**FIGURE 5:**
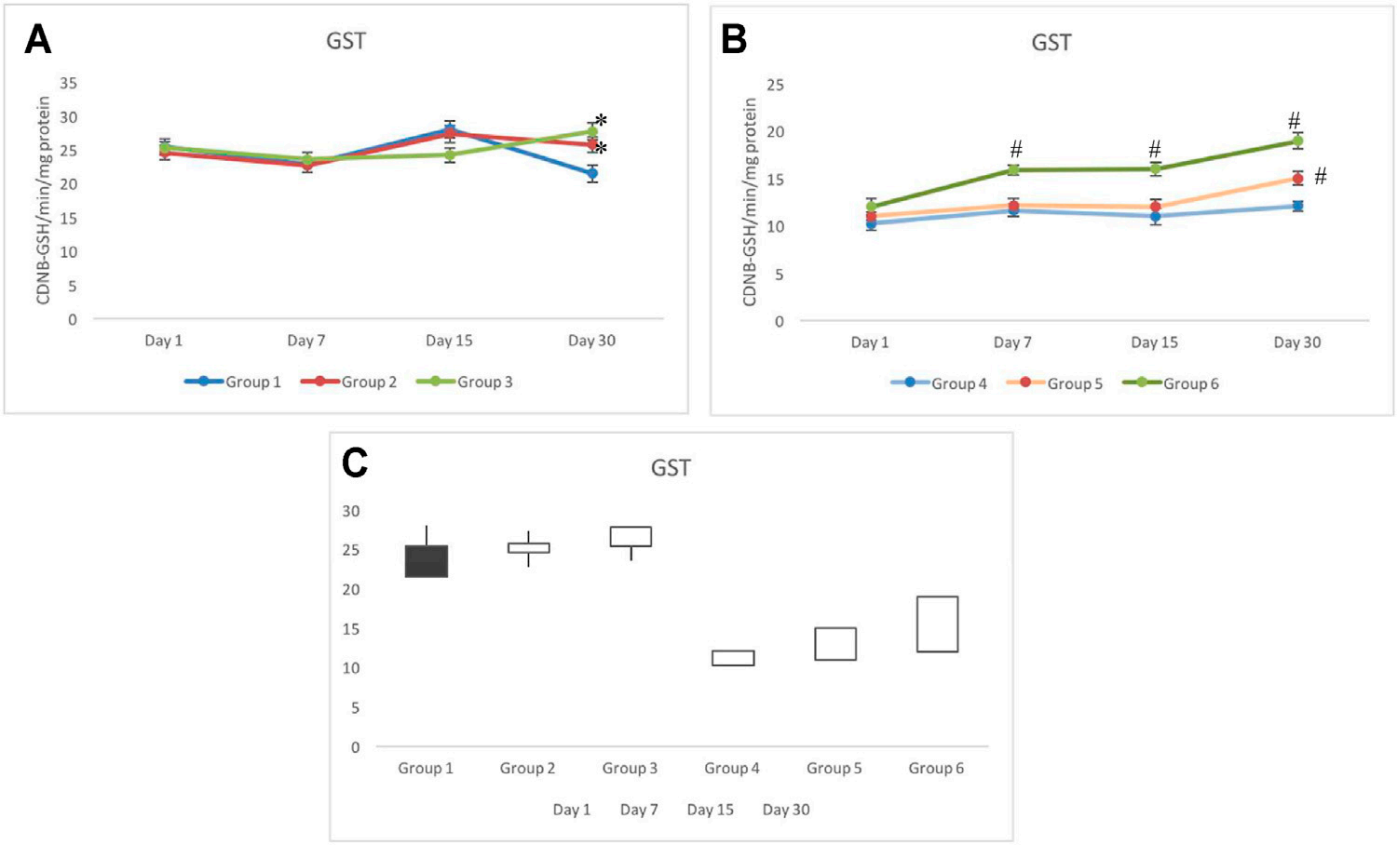
Animals pretreated with CRE/CRE-AgNPs indicated differential activity of glutathione-S-reductase (GST) in non-irradiated **(A)** and irradiated **(B)** groups. Change in activity from day 1 to day 30 was expressed in the box plot where black indicates the loss of activity and the white box indicates the gain in activity **(C)**. *p < 0.05 significance level was measured against group 1. ^**#**^ *p* < 0.05 significance level was measured against group 4.

Non-exposed groups 1 to 3 indicated a similar response for the GST activity in testicular tissues. The GST activity in group 1 ranged between 21 and 28 CDNB-GSH/min/mg protein during the period of investigation (i.e., days 1–30). On day 30, groups 2 and 3 indicated significant elevation in the activity of GST in comparison to group 1. Although an elevated activity was observed in groups 2 and 3 on day 30, it was still within the control range (Figure 5A). Irradiated groups 4 to 6 showed a distinct pattern in the activity of GST. Group 4 invariably showed a significantly lower activity throughout the days of investigation. On day 30, group 4 was measured with 12.07±0.54 CDNB-GSH/min/mg protein, which was at least two-folds lower than that of group 1. The GST response in group 5 was in parallel to the response of group 4 with slight elevation until day 15. On day 30, a significant elevation was observed, which was recorded as 14.99±0.72 CDNB-GSH/min/mg protein. However, the GST activity in group 6 was consistently higher during the periods of investigation (on days 1–30). Group 6 also noticed a gradual increase in the activity and was estimated as 18.85±0.72 CDNB-GSH/min/mg protein on day 30 (Figure 5B). The stock plot clearly indicated a maximum gain in the activity in group 6 followed by group 5 and group 3 (group 4C).

### 3.5 Glutathione Reductase

**FIGURE 6:**
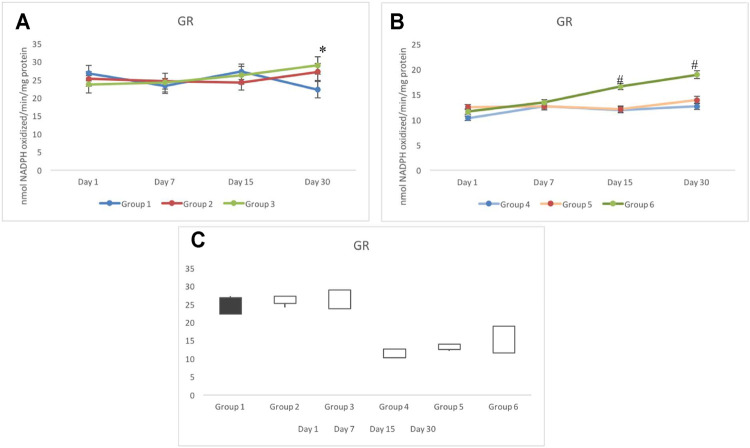
Activity of glutathione reductase (GR) in non-exposed **(A)** and exposed **(B)** animals pretreated with CRE/CRE-AgNPs. Gain and loss in activity is indicated with white and black boxes, respectively **(C)**. *p < 0.05 significance level was measured against group 1. ^**#**^ *p* < 0.05 significance level was measured against group 4.

No significant variation was observed in groups 1 to 3 for the GR response. The activity of GR in control testicular cells was noted in the range of 22 and 27 nmol NADPH oxidized/min/mg protein throughout the days of investigation (Figure 6A). In irradiated groups 4 to 6, a similar response of the GR activity was observed until day 7. In group 6, it uniquely elevated constantly from day 1 (measured weekly); however, a significant increase in the GR activity was observed from day 15 onward. GR activity in group 6 on day 15 and day 30 was estimated as 16.59±0.62 and 18.91±0.76 nmol NADPH oxidized/min/mg protein, respectively. Group 5 indicated similar GR response to that of group 4, which remained significantly lower than the control (group 1) (Figure 6B). The stock plot indicated a maximum gain in the activity of GR in group 6 followed by group 3 (Figure 6C).

### 3.6 Glutathione Peroxidase Response

**FIGURE 7:**
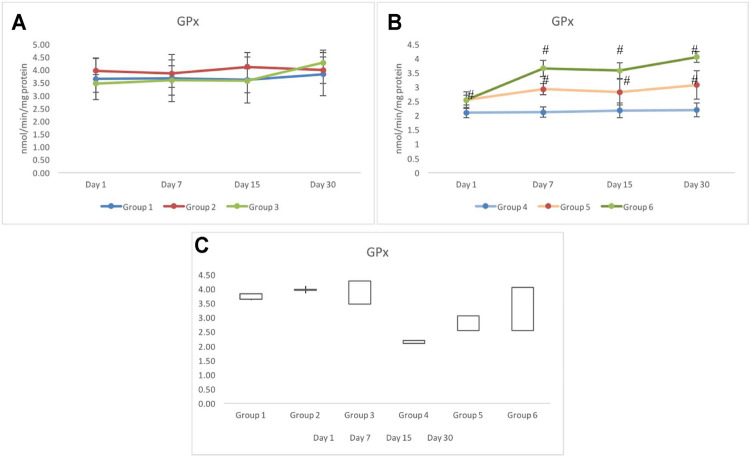
Variation in the activity of glutathione peroxidase (GPx) in non-exposed **(A)** and exposed **(B)** groups based on pretreatment with CRE/CRE-AgNPs. **(C)** indicating the stock plot indicating the gain/loss of activity on GPx from day 1 to day 30 of investigation. *p < 0.05 significance level was measured against group 1. ^**#**^ *p* < 0.05 significance level was measured against group 4.

The response of GPx activity in testicular tissues of non-exposed (groups 1–3) indicated a similar response. However, on day 30, a slightly higher activity in group 3 was observed, which was recorded as 4.27±0.49 nmol/min/mg protein. However, the GPx activity in group 1 was recorded as 3.83±0.84 nmol/min/mg protein on day 30 (Figure 7A). In exposed groups 4 to 6, a distinct differentiation in the GPx activity was observed. Where the maximum activity was observed in group 6, groups 4 and 5 indicated significantly lower and invariably similar values during all periods of investigation (i.e., days 1–30). A 50% decline in the activity of GPx was recorded in group 4, which remained stagnant throughout the period of investigation. Groups 5 and 6, however, showed better tolerance against irradiation with respect to group 4. Nevertheless, comparatively group 4 showed the minimal elevation as the days progressed from day 7 to day 30. The maximum activity of GPx was observed in group 6 on day 30, which was recorded as 4.05±0.19 nmol/min/mg protein; this activity was comparable to the control (group 1) (Figure 7B). Greatest gainer among all groups was group 6 followed by group 3 and group 5 (Figure 7C).

### 3.7 Catalase Response

**FIGURE 8:**
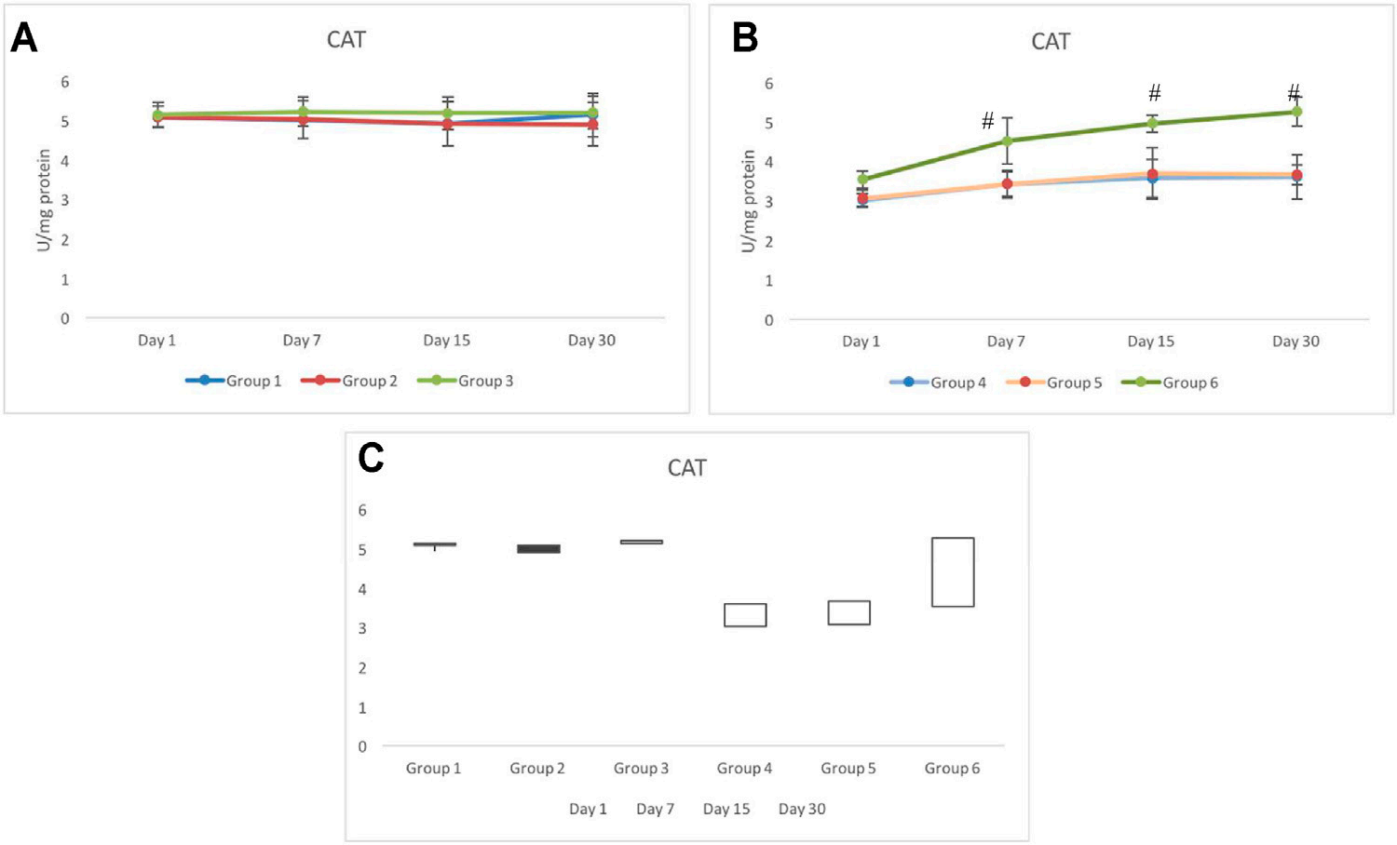
Response of catalase (CAT) in pretreated CRE/CRE-AgNP–treated non-exposed **(A)** and exposed **(B)** animals. Groups indicating the gain/loss in activity of catalase **(C)** during the duration of investigation. *p < 0.05 significance level was measured against group 1. ^**#**^ *p* < 0.05 significance level was measured against group 4.

Unlike any other antioxidant enzymes evaluated in this study, the CAT response for non-exposed groups 1 to 3 was distinctively different. The activity of CAT in groups 1 and 2 was similar until day 15. However, on day 30, group 1 indicated elevation in the activity of CAT (5.13±0.36 U/mg protein), whereas on day 30, the activity of CAT in group 2 further declined and was recorded as 4.91±0.55 U/mg protein. Group 3 consistently indicated a significantly higher activity of CAT than the control group (group 1) (Figure 8A).

Irradiated groups 4 to 6 indicated significantly lower activity of catalase on day 1 than the control group (group 1). Group 5 indicated a similar CAT response to that of group 4. There was no significant alteration in the activity of CAT from day 1 to day 30. However, the activity of CAT in group 6 not only tolerated irradiation better than group 4 and group 5 but also invariably elevated until day 30. The activity of CAT in group 6 was recorded as 4.52±0.58, 4.96±0.22, and 5.26±0.37 on day 7, day 15, and day 30, respectively. The activity of CAT in group 6 resumed back to the control range from day 7 onward (Figure 8B). The stock plot clearly showed that group 6 was the highest gainer of the activity of CAT, followed by group 4 and group 5 (Figure 8C).

### 3.8 Strength of Association Between Variations in the Activity of Antioxidant Enzymes

**FIGURE 9:**
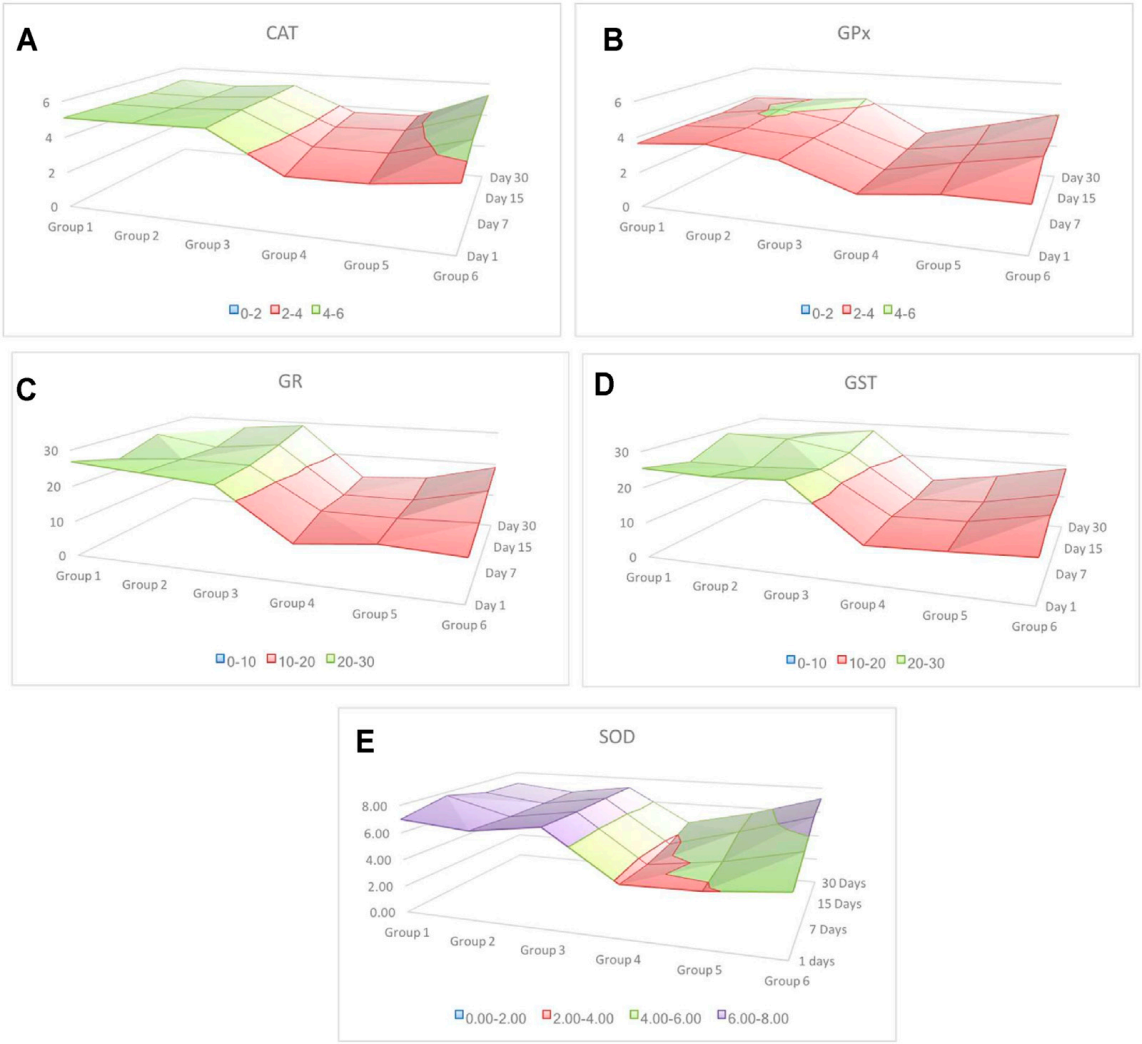
Surface plot indicating alteration in the activity of antioxidants from day 1 to day 30 of most cause-and-effect–related antioxidants. Irrespective of individual numeric values, different colors indicate group-wise distribution of variation. Red color indicates the lowest and purple the highest. The alteration in the activity of SOD is the highest compared to the rest of the four antioxidant responses. Among all, GPx showed the lowest variation, whereas GR and GST indicated a similar response.

Pearson’s correlation analysis indicated strong association between antioxidant enzymes. Strong association between antioxidant enzymes suggested synchronization against a common cause. LPO indicated negative correlation with all other antioxidants investigated in this study. However, the degree of association was still moderately strong. Among all associations, few indicated extremely high degree of associations, such as between GR and GST (r = 0.979), CAT and SOD (r = 0.954), and CAT and GPx (r = 0.909) (Table 1). Based on these observations, when a surface plot was drawn, it clearly showed that the common variable among CAT, SOD, and GPx was the performance of group 3 and group 6. With common ranges, CAT and GPx revealed a similar activity around days 15–30 for group 2 and group 3. Association between CAT and SOD does not reflect any obvious pattern; however, activities of these antioxidants appeared to be maximum and resumed back to control on day 30 of investigation, whereas activities of GR and GST for experimental groups 1 to 6 were strikingly similar to each other (Figures 9A–E).

**TABLE 1:** Strength of association between variables for individual antioxidant enzymes.

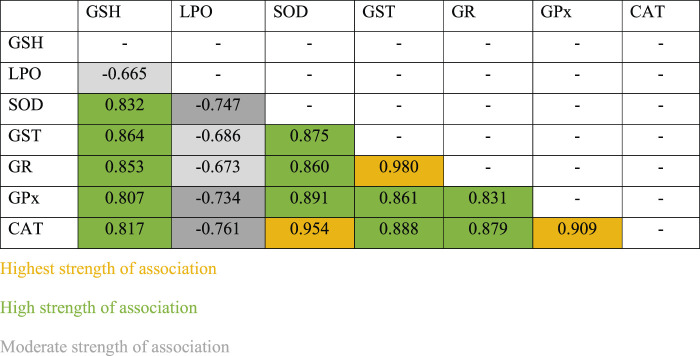

## 4 Discussion

The ancient Indian literature describes *C. borivilianum* with various names such as *Bhavaprakash nighantu* and *Rasendra Sarsangrah* (Grover, 2021). It is largely used as an ethnic medicine for the treatment associated with immunomodulation, immunostimulation, and as adaptogenic (Kirtikar and Basu, 1956; Triveni 1977; Sharma et al., 2001). A study by Thakur et al. (2007) examined this claim by administering the extracts on *Candida albicans*–infected albino rats. Results indicated increase in phagocytosis delayed-type hypersensitivity response and neutrophil adhesion. Authors observed a higher immunostimulant activity by *C. borivilianum* extract than sapogenins. There is an interlinking mechanism behind immunomodulation that is controlled by oxidative stress or *vice versa* (Ajith et al., 2017). Likewise, radiation leads to persistent oxidative stress that has implications with early and late effects of radiation (Datta et al., 2012). According to a study, nearly 40% of cancer patients receive at least 1 course of radiotherapy (Lalani et al., 2017). This technique still harbors both early and late side effects (Bentzen, 2006). To effectively mitigate radiation-related side effects, interventions toward a biological cascade must be performed in such a way that it does not lose its beneficial impact but simultaneously regulates its harmful effects on other tissues (Namoju et al., 2021).

This study hypothesized that if extracts of *C. borivilianum* have immunomodulatory effects, it should potentially regulate antioxidant enzymes. Therefore, an attempt was made to evaluate the differential expression of antioxidant enzymes in response to with and without radiation. This study also examined alterations in the bioavailability of the *C. borivilianum* root extract by tagging the extract particle with silver nanoparticles. Results indicated distinct differentiation between groups those were irradiated and those which were not. The study was observed for a maximum of 30 days, and the observations were made on the basis of expressions witnessed during four intervals, that is, day 1, day 7, day 15, and day 30. In most cases on day 1, the response of antioxidants in non-exposed groups was similar; however, in exposed groups, CRE-treated animals in groups 5 and 6 indicated slightly better toleration against radiation than those in group 4. Our previous study recorded lower histological damages in testicular architecture of those irradiated animals pretreated with the *C. borivilianum* root extract (Vyas et al., 2020). Thus, better toleration on day 1 was indicative of quicker resumption until day 30.

Reduced glutathione is one of the most abundant non-protein thiols and present in all mammalian tissues. A review by Bump and Brown (1990) mentioned that radiation induces self-destructive damages in tissues, and most of such damages were initiated by oxidative radicals. Authors suggested that following radiation competing reactions were very rapid, thus requiring high concentrations of antioxidant enzymes. Under hypoxic conditions, the availability of GSH highly affects the radiosensitivity. Our results indicate that concentration of GSH in testicular tissues was better from day 1 itself. Besides the increase in the rate of GSH biosynthesis, a constant weekly elevation in the level of GSH was evident in both CRE- and CRE-AgNP–administered groups. The level of GSH was higher in CRE-AgNP–administered animals, which was indicative of higher bioavailability. It is to be noted that nanonization provides enhanced bioavailability and therapeutic effectiveness (Jia, 2005).

A xenobiotic substrate presenting GST activity is important in understanding the resulting GSH level in testicular tissues. This study noted that animals pretreated with CRE and CRE-AgNPs responded with better resumption in the activity of GST than irradiated animals not treated with the *C. borivilianum* root extract. The activity of GST is dependent on the availability of GSH in the testicular tissues. Therefore, observations for the group-wise activity of GST are completely justified with the level of GSH observed in CRE- and CRE-AgNP–treated animals. Likewise, GR is an important enzyme for recycling GSH from glutathione disulphide (GSSG) (Deponte, 2013). The activity of GR can effectively regulate the level of GSH in the testicular cells. Results of this study indicate that the activity of GR in exposed groups was variably dependent on the type of treatment given before irradiation. Exposed animals treated with CRE-AgNPs indicated maximum recovery on day 30 of investigation followed by CRE treatment and non-treatment. The maximum level of GSH in CRE-AgNP–treated animals is justified as the activity of GR was also the highest among other groups.

Another important enzyme in GSH recycling and simultaneous reduction of lipid hydroperoxides and hydrogen peroxides is GPx. The activity of GPx decides production of GSSG, a precursor to GSH (Bhabak and Mugesh, 2010; Muthukumar et al., 2011). For cells to have abundant GSH, the presence of GSSG and GR is critical. This study indicates the reciprocal activity of GPx. CRE-treated animals indicated a significantly higher activity of GPx than exposed non–CRE- or CRE-AgNP–treated animals. The maximum activity of GPx was observed in exposed animals, pretreated with CRE-AgNPs, which corresponded to the availability of GSH in testicular cells. Despite distinct differences in the response of CRE-treated animals following irradiation, there was a similar response of glutathione-related enzymes in non-exposed groups. It was confirmed that the administration of the *C. borivilianum* root extract maintains homeostasis of GSH and GSH-related enzymes. In response to the gamma radiation, a substantial increase in the level of GSH and related enzymes, that is, GST, GR, and GPx, were observed, with respect to the sham control.

Lipid peroxidation is a process where lipid molecules lose electrons to free radicals, leading to further downstream reaction(s) causing damage to the plasma membrane. A detailed study by Kiang et al. (2012) proposed that LPO is one of the main causes of cell death following ionizing irradiation. Our study indicated that animals which were administered with CRE and CRE-AgNPs had lower LPO levels than irradiated animals not treated with CRE. A study by Govindarajan et al. (2005) reported a lower level of LPO in male Sprague–Drawley rat administration with the *C. borivilianum* extract. A gradual decline in the level of LPO was observed in this study following day 1 to day 30. However, it was striking that irradiated animals those that were not pretreated with the *C. borivilianum* root extract also rapidly revived, indicating the maximum depletion in LPO levels following irradiation. Previous studies have noted rapid adjustment of antioxidants following a short-term exposure to radiation (Borek, 2004; Okunieff et al., 2009).

Superoxide dismutase is an important enzyme for dismutation of superoxide radicals. Isoforms of SODs such as EcSOD and MnSOD have been studied widely for their antitumor and redox homeostasis effects (Griess et al., 2017; Ighodaro and Akinloye, 2018). SOD is considered radioprotectant. If SOD is injected intravenously hours before whole body radiation, it can significantly reduce the impact of irradiation (Petkau and Chelack, 19784). Post-irradiation, the activity of SOD can decide the amount of damage triggered through radiation. This study noted a distinct level of activity between exposed animals pretreated with CRE, CRE-AgNPs, and those without treatment. This study also indicated the lowest activity in radiation-alone groups, whereas animals pretreated with CRE-AgNPs indicated a maximum activity. Animals pretreated with CRE also revealed better recovery through all days of investigation. A study by Giribabu et al. (2014) reported that activity of SOD increased and resumed back to normal in CRE-treated diabetic rats with proven depletion in the activity of SOD. Nonetheless, in this study, the activity of SOD did not resume back to the control range, except for CRE-AgNP–treated animals. Animals pretreated only with CRE remained significantly lower than control animals. The difference in observation of CRE-led SOD resumption in diabetic rats and irradiated mice could be due to the type of oxidative stress generated by individual conditions. Where oxidative stress in diabetes is generated through metabolic abnormalities and mitochondrial overproduction of superoxide (Giacco and Broeniee, 2010), ionizing radiation causes radiolysis of water molecules, leading to extreme surge of superoxide radicals (Datta et al., 2012).

Catalase is not only the most common endogenous antioxidant enzyme in living cells but also has the highest turnover numbers. A study by Xiao et al. (2015) estimated radiation-induced apoptosis in hematopoietic stem cells and progenitor cells. Authors revealed that CAT treatment markedly inhibits irradiation-induced apoptosis in hematopoietic stem cells. Previous studies have noted that overexpression of CAT can protect tissues against oxidative insults (Shen et al., 2012; Liao et al., 2013; Miao et al., 2013). The present study is in accordance with the above reports, and results indicate a significant increase in the level of CAT in those animals pretreated with CRE-AgNPs. However, CRE-treated animals responded similar to that of the irradiation-alone group. Unlike other antioxidants, administration of CRE alone was not significantly effective in increasing the activity of CAT following irradiation. It was not understood why the efficacy of CRE was lower than that of CRE-AgNPs, and it was hypothesized that AgNPs or the size of CRE particles may have contributed in altering the CAT expression.

Oxidative stress and antioxidant enzymes have significant roles in the functioning of reproductive system. Testicular tissues consist of various nursing cells, germ cells, and spermatozoa; the functional performance of which defines fertility success. The complex process of spermatogenesis requires adequate number of antioxidants to protect against sperm dysfunction. According to a study, nearly 30–40% of infertile men have elevated levels of ROS in their reproductive system (Lanzafame et al., 2009). Another study by Maiorion and Ursini (2002) explained that ROS production and depletion of glutathione in the germ cell are prerequisites for functional maturation and capacitation of spermatozoa. They further stressed that the role of hydrogen peroxides, selenium, and oxidation of protein sulfhydryl groups during the last stage of spermatogenesis is extremely crucial.

Irradiation affects the process of spermatogenesis greatly, and it causes azoospermia in those who receive higher doses (Okada and Fujisawa, 2019). Although sperm count resumes back to normal, it may take as long as 2 years following irradiation (Meistrich and van Beek, 1990). This study explored cause and effect variation between each antioxidant investigated, and revealed that antioxidants such as CAT and SOD, GR and GST, and CAT and GPx are extremely associated. Though association between GR and GST is understood, as both antioxidants are complementary to the recirculation of GSH, strength of association between CAT-SOD and CAT-GPx is noteworthy. Many studies have reported that SOD, GPx, and CAT are the main endogenous enzymatic defense systems in all aerobic cells (Halliwell and Gutteridge, 2007; Fisher-Wellman et al., 2009). A previous study indicated that these three antioxidants provide protection by scavenging superoxide directly (Milic et al., 2009). Although statistically these three antioxidants are likely to be interconnected, the surface plot showed a pattern in the activities of enzymes in response to two distinct variables, that is, CRE/CRE-AgNPs and irradiation. This indicated highest alteration in SOD followed by CAT and GPx. It justifies the general antioxidant protocol, which describes that SODs convert superoxide into H_2_O_2_ and molecular oxygen, whereas CAT and GPx convert H_2_O_2_ into water (Weydert and Cullen, 2010). Therefore, this study confirmed a coordinated elevation in the activity of antioxidants following irradiation in those animals treated with CRE and CRE-AgNPs.

Thus, this study shows that CRE-AgNPs have an edge over the *C. borivilianum* root extract alone against irradiation exposure. We used a comparatively low dose of the *C. borivilianum* root extract and CB-AgNPs in this study and received some unanticipated results from animals administered with CRE-AgNPs. The overall performance of CRE-AgNPs was equivalent to results of earlier studies which used daily doses as high as 800 mg/kg body weight. We are aware that nano-molecules have greater bioavailability and are able to cross cellular barriers without being metabolically inert or excreted. With CRE-AgNP mice exposed to irradiation, results showed improved retaliation and the rate of improvement through period of investigations. Therefore, biosynthesis of nanoparticles not only increases their efficacy and bioavailability but also reduces the dose required; thereby, it can also be helpful in replenishing the loss of natural biodiversity of *C. borivilianum.* Exposure to radiations either ionizing or non-ionizing may have several harmful effects on the brain and the reproductive system (Kesari et al., 2013; Kesari et al., 2018). However, the use of plant-derived biomolecules or phytochemicals especially from Indian ethnomedicines may play a major role in the treatment of severe diseases (Kesari et al., 2020; Fatima et al., 2021) which may be caused by mutagenic factors (chemical, physical, and biological). Moreover, this study not only establishes CRE and CRE-AgNPs as radioprotectors but also opens the door for more detailed research on CRE-AgNPs and whether they can be exploited for pharmacological properties like antioxidant, antidiabetic, antitumor, or aphrodisiac.

Furthermore, the present study confirmed that antioxidant enzymes in non-irradiated groups mostly remained under the control range. However, after irradiation, these enzymes performed better against induced oxidative stress in testicular tissues of those animals treated with CRE or CRE-AgNPs. In irradiated groups, a slightly better toleration on day 1 was observed. By day 30, most antioxidants elevated significantly as compared to day 1. However, the elevation was extremely maximum in those animals treated with CRE-AgNPs. Although CRE has differential expression of antioxidants post-irradiation, the effects were limited. Nonetheless, it can be concluded affirmatively that CRE-AgNPs have distinct overexpression of antioxidants following irradiation-induced oxidative stress.

## Data Availability

The original contributions presented in the study are included in the article/Supplementary Material, further inquiries can be directed to the corresponding authors.
